# Pierisin, Cytotoxic and Apoptosis-Inducing DNA ADP-Ribosylating Protein in Cabbage Butterfly

**DOI:** 10.3390/toxins16060270

**Published:** 2024-06-14

**Authors:** Azusa Takahashi-Nakaguchi, Yu Horiuchi, Masafumi Yamamoto, Yukari Totsuka, Keiji Wakabayashi

**Affiliations:** 1 Medical Mycology Research Center, Chiba University, 1-8-1 Inohana, Chuo-ku, Chiba 260-8673, Japan; 2 Aquatic Food Research Laboratory, Central Research Institute, Tokyo Innovation Center, Nissui Corporation, 1-32-3 Shichikoku, Hachioji City 192-0991, Japan; 3 Central Institute for Experimental Medicine and Life Science, 3-25-12 Tonomachi, Kawasaki-ku, Kawasaki 210-0821, Japan; 4 Department of Environmental Health Sciences, Hoshi University, 2-4-41 Ebara, Shinagawa-ku, Tokyo 142-8501, Japan; 5 Graduate Division of Nutritional and Environmental Sciences, University of Shizuoka, 52-1 Yada, Suruga-ku, Shizuoka 422-8526, Japan

**Keywords:** Pierisin-1, DNA ADP-ribosylation, *Pieris rapae*

## Abstract

Pierisin-1 was serendipitously discovered as a strong cytotoxic and apoptosis-inducing protein from pupae of the cabbage butterfly *Pieris rapae* against cancer cell lines. This 98-kDa protein consists of the N-terminal region (27 kDa) and C-terminal region (71 kDa), and analysis of their biological function revealed that pierisin-1 binds to cell surface glycosphingolipids on the C-terminal side, is taken up into the cell, and is cleaved to N- and C-terminal portions, where the N-terminal portion mono-ADP-ribosylates the guanine base of DNA in the presence of NAD to induce cellular genetic mutation and apoptosis. Unlike other ADP-ribosyltransferases, pieisin-1 was first found to exhibit DNA mono-ADP-ribosylating activity and show anti-cancer activity in vitro and in vivo against various cancer cell lines. Pierisin-1 was most abundantly produced during the transition from the final larval stage to the pupal stage of the cabbage butterfly, and this production was regulated by ecdysteroid hormones. This suggests that pierisn-1 might play a pivotal role in the process of metamorphosis. Moreover, pierisin-1 could contribute as a defense factor against parasitization and microbial infections in the cabbage butterfly. Pierisin-like proteins in butterflies were shown to be present not only among the subtribe Pierina but also among the subtribes Aporiina and Appiadina, and pierisin-2, -3, and -4 were identified in these butterflies. Furthermore, DNA ADP-ribosylating activities were found in six different edible clams. Understanding of the biological nature of pierisin-1 with DNA mono-ADP-ribosylating activity could open up exciting avenues for research and potential therapeutic applications, making it a subject of great interest in the field of molecular biology and biotechnology.

## 1. Discovery and Characterization of Pierisin-1

Dr. Takashi Sugimura was a major international figure in cancer research. He was appointed as president of the National Cancer Center, Tokyo, in 1984 and became president emeritus in 1992.

Dr. Sugimura has had a strong interest in insects, especially butterflies, since his boyhood. By some chance, our research group including Dr. Sugimura and the authors in this review at the National Cancer Center Research Institute, Tokyo, discovered the existence of a biologically very interesting protein, named “pierisin”, in the butterfly, and in this review, we would like to explain how it came about and what we have achieved.

**Figure 1:**
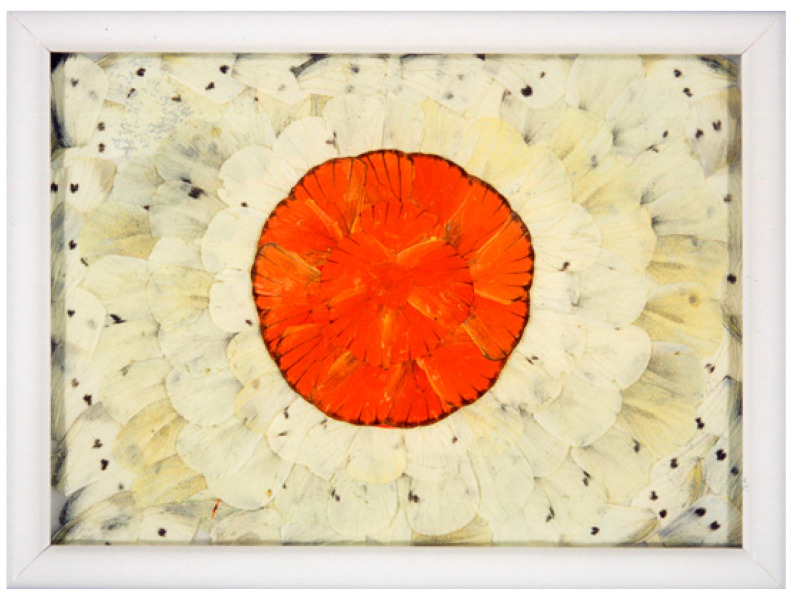
Handmade butterfly picture of the national flag of Japan. The central red circle on the flag was made from the red wings of *Appias nero*, and the white portion was made from the common cabbage butterfly, *Pieris rapae*.

**Figure 2:**
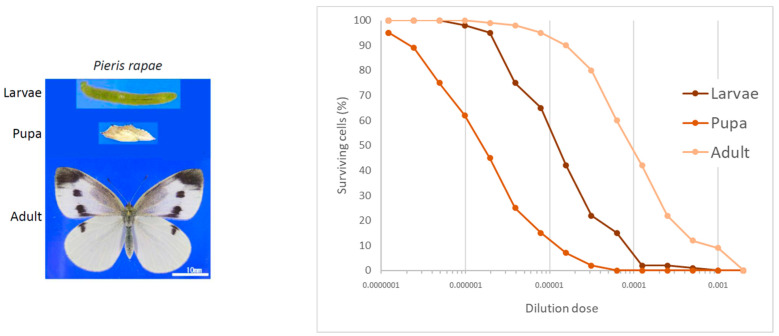
Cytotoxic effects of extracts from the larvae, pupae, and adults of *P. rapae*. Extracts from larvae, pupae, and adults were incubated at various dilutions with TMK-1 cells and after 48 h incubation at 37 °C in 5% CO_2_ in air. The number of living cells was measured with XTT cell proliferation assay [2].

In 1997, Dr. Sugimura and Dr. Bruce Ames (University of California) jointly received the Japan Prize for the “Establishment of the Concept of Genetic Alterations for Cancer Development”. To celebrate the occasion, Dr. Koutaro. Koyama, former researcher at our research institute and a butterfly enthusiast, presented Dr. Sugimura with a handmade butterfly picture of the national flag of Japan, made of butterfly wings. The central red circle on the flag was made from the red wings of *Appias nero* from Southeast Asia, and the white portion was made from the common cabbage butterfly, *Pieris rapae* (Figure 1). The price of *A. nero* obtained through a dealer was so expensive, and the size of the butterfly picture had to be reduced. This meant that many larvae, pupae, and adults of cabbage butterfly, which were collected from a cabbage farm in the suburb areas of Tokyo and Yokohama city in Japan, were superfluous; however, to throw them away would have been very wasteful. Dr. Sugimura came up with the idea of incubating their extracts with TMK-1 cells [1], a human gastric cancer cell line that had been cultured in our laboratory because the larvae, pupae, and adults of cabbage butterfly must contain some active principles, which are involved in self-defense systems and metamorphosis and induce some changes in TMK-1 cells. Surprisingly, and contrary to everyone’s expectations, the pupae extract showed strong cytotoxic activity. Nuclear fragmentation, chromatin condensation, and DNA fragmentation were also observed, indicating that apoptosis was induced in TMK-1 cells [2]. Cytotoxic activity was also observed in larvae and adults, but the activity was strongest in pupae, followed by larvae and adults (Figure 2). The results were more than enough to induce a spirit of inquiry as to why such an active principle exists in the cabbage butterfly.

**Figure 3:**
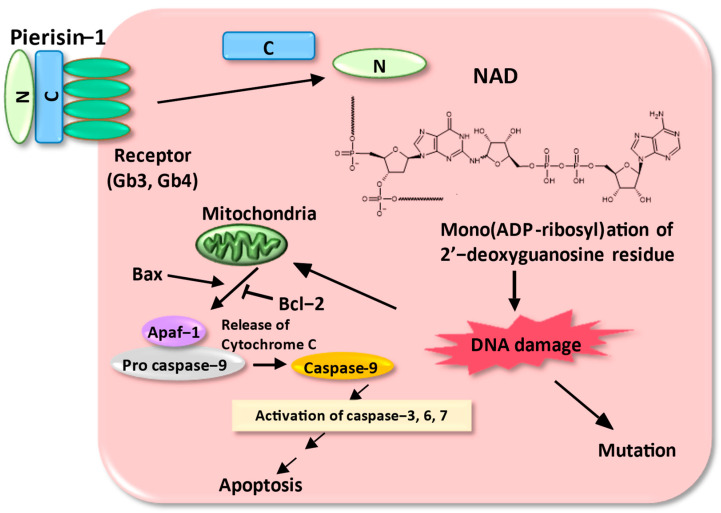
The apoptosis and mutation induction mechanism of pierisin-1 in mammalian cells. Pierisin-1 binds to the receptors Gb3 and Gb4, which are present in mammalian cells and is internalized into the cell. Inside the cell, it separates into N-terminal and C-terminal fragments and mono-ADP-ribosylates the guanine of DNA. This DNA adduct causes DNA damage, leading to apoptosis through the mitochondrial pathway involving Bcl-2.

The active principle in the pupae of *P. rapae* was heat-labile, precipitable with ammonium sulfate and inactivated by protease, suggesting that it is a protein. This cytotoxic active principle was isolated and purified from large quantities of pupae of cabbage butterfly using various column chromatographies, and the cytotoxic principle was a 98-kDa protein, named pierisin-1 [3]. Sequencing and cloning of a cDNA encoding pierisin-1 showed that the gene encodes an 850 amino acid protein with a calculated molecular weight of 98,081. The N-terminal portion of pierisin-1 contains a sequence homology with bacterial toxins with ADP-ribosylation activity such as mosquitocidal toxin [4,5,6,7,8], Diphtheria toxin [9,10,11,12], and cholera toxin [13], and the C-terminal portion shows similarity to HA-33, a subcomponent of hemagglutinin of botulinum toxin [14,15]. Initially, we thought that the target molecule for the ADP-ribosylation of pierisin-1 was a protein or amino acid substrate, like other ADP-ribosyltransferases, but we could not find a hit molecule. A mixture of [^32^P] NAD, Pronase-treated pierisin-1, and cell-free extracts from HeLa cells, which are highly sensitive to pierisin-1, was incubated, and the incorporation of radioactivity was carefully analyzed using SDS/PAGE followed by autoradiography. Pronase treatment produced “nicked” pierisin-1. It appears to consist of properly associated N- and C-terminal fragments with a similar structure to that of intact pierisin-1. The pronase-treated pierisin-1 transferred 10-fold more radioactivity from [^32^P] NAD to cell extracts than untreated pierisin-1. The majority of the radioactivity was recovered in high-molecular-weight material fractions, and these radioactive fractions were found to be digested with DNase but not protease. These results suggest that DNA may be the acceptor for ADP-ribosylation by pierisin-1. Indeed, pierisin-1 was shown to efficiently catalyze the ADP-ribosylation of double-stranded DNA containing dG-dC but not dA-dT pairs, and it was found to react specifically with the N-2 position of the guanine base in DNA. Namely, pierisin-1 incubated with DNA and β-NAD resulted in the formation of N2-(ADP-ribos-1-yl)-2′-deoxyguanosine (Figure 3) [16].

The function of the N- and C-terminus of pierisin-1 was examined for cytotoxic activity against human cervical carcinoma HeLa cells using peptides synthesized in vitro from cDNA with the rabbit reticulocyte lysate system. As a result, it was shown that pierisin-1 is a cytotoxic protein that binds to receptors on the cell membrane at the C-terminal region, and after being taken up into the cell, the enzyme domain in the N-terminal region mono-ADP-ribosylates the target molecular DNA in the cell [17]. The three-dimensional structure of the N-terminal region of pierisin-1 was clarified through collaborative research with Dr. Mamoru Sato’s laboratory at Yokohama City University. Based on the crystal structures of the catalytic domain of pierisin-1 and its mutation analysis, β-NAD binding was shown to occur in a manner similar to that of the mono-ADP-ribosylating bacterial toxins, with the phosphate–nicotinamide loop and basic cleft playing important roles in DNA binding [18].

The C-terminal peptide of pierisin-1 has an amino acid sequence homologous to HA-33, a subcomponent of hemagglutinin of botulinum toxin [14,19,20,21,22,23,24]. Cross-linking and cloning experiments were performed to identify receptors for pierisin-1. These results suggested that proteins on the cell membrane had no binding ability to pierisin-1. Inhibitory assays of lipids fractionated from HeLa cells demonstrated that neural glycosphingolipids on the cell surface exhibit receptor activity. Mass spectrometry and antibody experiments revealed that the receptors for pierisin-1 in HeLa cells are globotriaosylceramide (Gb3) and globotriaosylceramide (Gb4) [25]. It is known that Shiga toxin also has a receptor with Gb3 as the ligand [26]. However, the QXW sequence in the lectin domain of the lysine B chain, common to pierisin-1 and HA33, is absent in Shiga toxin, indicating a different recognition mechanism [27].

The apoptosis pathway induced by pierisin-1 was triggered by DNA ADP-ribosylation, and this DNA damage was shown to be mainly mediated by the mitochondrial pathway involving Bcl-2 [28]. Furthermore, pierisin-1 induced mutations in the HPRT (hypoxanthine-guanine phosphoribosyltransferase) gene in CHL cells in a dose-dependent manner, with activity 10,000-fold stronger than that of a typical carcinogen MNNG (N-methyl-N’-nitro-N-nitrosoguanidine) [29,30,31,32,33,34,35]. The mutational spectrum of this HPRT gene by pierisin-1 was dominated by G to C and G to T transversions which occurred in the contexts 5′-tgga-3′ or 5′-tggt-3′ [36].

To summarize these results, pierisin-1 binds to cell surface glycosphingolipids Gb3 and Gb4 on the C-terminal side, is taken up into the cell by endocytosis, and is degraded by intracellular proteases into N- and C-terminal portions, where the N-terminal portion ADP-ribosylates the guanine base of DNA in the presence of NAD. This DNA damage is thought to trigger cellular genetic mutation and apoptosis (Figure 3) [37,38].

The purified pierisin-1 was tested for its inhibitory activity against more than 10 cancer cell lines. Pierisin-1 showed cytotoxic effects in all cell lines, and the most sensitive cell line was HeLa cells with an IC50 of 0.043 ng/mL. In contrast, the mouse melanoma cell line was less sensitive, with an IC50 of 270 ng/mL, a difference of approximately 6000-fold from the sensitivity of HeLa cells [39]. Furthermore, the HeLa cells were inoculated intraperitoneally into 6-week-old female nude mice and intraperitoneally injected 24 h after pierisin-1 at a concentration of 3 μg/kg. Thereafter, the mice were sacrificed at day 80. The mean tumor weight was significantly reduced with pierisin-1 treatment, and anti-cancer activity in vivo was demonstrated [40]. The LD50 of pierisin-1 was found to be ~5 μg/kg when pierisin-1 was intraperitoneally injected into the mice [41], showing strong toxicity comparable to that of typical toxic substances such as Diphtheria toxin [9,10,11,12], *Pseudomonas* exotoxin [42,43,44,45], and ricin [46,47].

## 2. Distribution of Pierisin-like Proteins in Various Kinds of Butterflies

To determine the distribution of pierisin-like proteins in butterflies, crude extracts from 20 species of the family Pieridae were examined for cytotoxicity in HeLa cells and DNA ADP-ribosylating activity [48,49,50]. Pierisin-like proteins have been suggested to be present in the extracts from butterflies not only among the subtribe Pierina but also among the subtribes Aporiina and Appiadina. Coincidentally, we found that pierisin-like protein was also present in *A. nero*, the wings of which were used for the central red circle on the butterfly picture of the national flag of Japan. We performed the cDNA cloning of pierisin-like proteins, pierisin-2 from another cabbage butterfly, *Pieris brassicae*, pierisin-3 from gray-veined white, *Pieris melete*, and pierisin-4 from black-veined white, *Aporia crataegi*. The nucleotide sequences of pierisin-2, -3 and -4 encode an 850, 850, and 858 amino acid protein, respectively. The deduced amino acid sequence revealed that pierisin-2 is 91% similar to pierisin-1, pierisin-3 is 93% similar, and pierisin-4 is 64% similar. These three proteins synthesized in vitro with the rabbit reticulocyte lysate exhibited apoptosis-inducing activity against HeLa and TMK-1 cells. Moreover, pierisin-2, -3, and -4 incubated with DNA and β-NAD resulted in the formation of N2-(ADP-ribos-1-yl)-2′-deoxyguanosine, as in the case of pierisin-1 [51,52,53]. Another research group reported that apoptosis-inducing pierisin-5 and pierisin-6 genes were identified and characterized from cabbage butterfly, *Pieris canidia*, and *Pieris napi*, respectively [54,55].

## 3. Biological Role of Pierisin-1 in Cabbage Butterfly

In order to clarify the biological role of pierisin-1 in the cabbage white butterfly, its expression during developmental stages, including the larva, pupa, and adult stages, was examined. Low levels of pierisin-1 mRNA and protein were detected in first-instar larvae, and their levels were increased around 5–100 times from the first-instar larvae to the fifth-instar larvae and then decreased by over 90% during the pupal and adult stages. Immunostaining of pierisin-1 demonstrated the protein to be mainly located in the fat bodies of fifth-instar larvae and early-phase pupae. Thus, pierisin-1 was most abundantly produced during the transition from the final larval stage to the pupal stage, a stage where apoptosis, or programmed cell death, occurs most frequently in insect metamorphosis [56,57].

**Figure 4:**
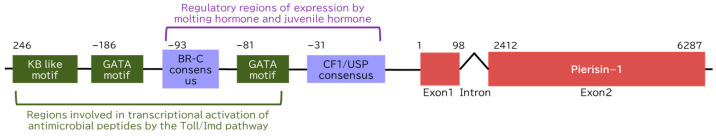
Transcriptional regulatory motifs: Several transcriptional regulating motifs, including BR-C (Broad Complex), CF1/USP (Cockerell Factor 1/Ultraspiracle), κB-like motifs, and GATA motifs, were found in close proximity to the transcription start site of the pierisin-1 gene. BR-C is known to control the response to ecdysone by binding to the promoter regions of target genes. CF1/USP forms heterodimers with ecdysone receptors and binds juvenile hormones. κB-like motifs are recognized by the κB family proteins for transactivation and are commonly present in the regulatory regions of many antimicrobial protein genes in insects. GATA motifs are also closely situated in the regulatory regions of numerous immunity genes.

The promoter region of pierisin-1 contains a region that regulates the activation of the metamorphic hormones (Figure 4) [58]. Actually, the production of pierisin-1 was found to be regulated by ecdysteroid hormones (Figure S1), which play a central role in controlling insect metamorphosis, as well as juvenile hormones [59,60]. This suggests that pierisin-1 might play a pivotal role in the process of metamorphosis. Pierisin-1 is produced in the fat body, equivalent to the mammalian liver, and released into the hemolymph (insect blood). It was further observed that pierisin-1 migrated to tissues, such as the midgut, that undergo apoptosis during the metamorphic period [61] (Figure S2). Gb3 and Gb4 are known to be widely present in mammals, but their presence in insects has not been confirmed. In preliminary experiments, neutral glycolipids from *P. rapae* were subjected to TLC, and pierisin-1 was shown to bind to these glycolipids using pierisin-1 antibodies. It was observed that pierisin-1 bound to a glycolipid in *P. rapae* that was larger in size than Gb3. This glycolipid is likely to be a receptor in *P. rapae* cells, but further verification is required.

While other insects undergo apoptosis even without pierisin-1 [56,62,63,64], it raises questions about whether only pierisin-1 possesses the functional activity of apoptosis in cabbage white butterfly. If the expression of the pierisin-1 gene is suppressed, would the metamorphosis into a pupa be halted? Recently, the entire genome of the cabbage white butterfly was elucidated [63,65,66], and significant advancements have been made in insect RNAi technology [67,68,69,70,71]. Leveraging these cutting-edge technologies will be crucial for conducting further analyses to fully understand how pierisin-1 contributes to apoptosis during metamorphosis in the cabbage white butterfly.

Another possibility is that the strong cytotoxicity of pierisin-1 might be effective as a protective agent against parasitoids and/or microbes. Therefore, attention was directed toward parasitoid wasps that thrive in the bodily fluids of cabbage white larvae, where pierisin-1 is abundant. These parasitoid wasps puncture the larvae’s body and emerge just before pupation. Experimental results revealed that parasitoid wasps that have evolved to specifically parasitize cabbage white butterflies possess resistance to pierisin-1. In contrast, pierisin-1 caused strong detrimental effects on eggs and larvae of closely related parasitoid wasp species [72]. The surface structure of eggs and larvae from parasitic wasps that are resistant to pierisin-1 prevents the toxin from entering their bodies, which is an important defense mechanism because the cells themselves remain sensitive to pierisin-1. Thus, it is suggested that pierisin-1 could contribute as a defense factor agent against parasitization by some types of wasps in the cabbage white butterfly.

Furthermore, the cabbage white butterfly, like mammals, is susceptible to bacterial and fungal infections. We examined the effectiveness of pierisin-1 against microbes including bacteria and fungi (Table S1). Pierisin-1 showed potent cytotoxic activity against fungi, especially against *Candida albicans*, while the bacteria cytotoxic effect of pierisin-1 was relatively low when tested against bacteria. However, when bacteria (*Micrococcus luteus* or *Escherichia coli*) were injected into the first day of third-instar larvae of cabbage white butterfly, induction of pierisin-1 expression was observed in the fat body or hemocytes (Figure S3). In fact, regions that are involved in the transcriptional activation of insect antimicrobial peptides were identified in the promoter region of pierisin-1 (Figure 4). These findings led us to consider the possibility that the regulation of pierisin-1 expression may also be utilizing conventional immune activation pathways. Thus, a multifaceted role for pierisin-1 was suggested in host defense mechanisms.

## 4. Distribution of Pierisin-like Proteins in Other Species Than Butterflies

To study the biological importance of DNA ADP-ribosylation, we attempted to identify the distribution of DNA ADP-ribosylating activities in various species, including insects, fish, and mammals. During this screening, we identified ADP-ribosylation activity targeting the N-2 position of the guanine base in DNA in six different edible clams, including *Meretrix lamarckii*, *Ruditapes philippinarum*, and *Corbicula japonica*. We purified the DNA ADP-ribosylating protein in the hard clam *M. lamarckii*, designated as CARP-1, and cloned its cDNA. The cDNA encodes a 182-amino acid protein with a calculated molecular mass of 20,332. CARP-1 in the culture medium exhibited no cytotoxicity against HeLa and TMK-1 cells. However, the introduction of this protein via electroporation induced apoptosis in these cells. These results suggest that CARP-1 is an ADP-ribosyltransferase without a receptor-binding domain [73,74].

Subsequently, SCO5461 in the *Streptomyces coelicolor* A3(2) was found to produce N(2)-(ADP-ribos-1-yl)-guanosine when co-incubated with guanosine and NAD(+). SCO5461 ADP-ribosylated various guanosine-related compounds and biomolecules and designated the SCO5461 protein product as *S. coelicolor* ADP-ribosylating protein, ScARP [75]. It is also reported that specific, reversible ADP-ribosylation of DNA on thymidine bases occurred in cellulo through the DarT-DarG toxin-antitoxin system in a variety of bacteria [76]. Furthermore, the molecular basis was elucidated, showing that the toxin DarT1 links ADP-ribose to the amino group of guanines in ssDNA, and NADARs function as antitoxins by reversing DarT1-catalyzed guanine ADP-ribosylation [77]. While the presumed cellular target still awaits final experimental proof, an ADP-ribosylating AB-toxin, Plx1, among the pathogenic factors of Paenibacillus larvae, a Gram-positive spore-forming bacterium notorious for causing American foulbrood, shares significant similarity with pierisin-1 [78,79,80,81]. Moreover, the mechanism for target specificity of protein- and DNA-targeting ADP-ribosyltransferases was demonstrated, revealing common mechanisms of target residue specificity among both protein- and DNA-targeting ARTs [82,83].

## 5. Future Perspectives

ADP-ribosylation is known to be a post-translational modification in which the ADP-ribose moiety of *β*-NAD is transferred to specific proteins. Several bacteria have been shown to produce mono(ADP-ribosyl)transferase, the acceptors of which are usually specific amino acid residues in proteins in eukaryotic cells. Cholera toxin and pertussis toxin ADP-ribosylate arginine and cysteine residues in G proteins, respectively. Diphtheria toxin modifies the diphthamide of elongation factor-2. Clostridium botulinum C3 exoenzyme is an asparagine-specific ADP-ribosyltransferase. There are also reports of mono-ADP-ribosyltransferases in mammals and avian species. Thus, mono(ADP-ribosyl)ation reactions occur at nitrogen or sulfur atoms in different amino acids [84,85].

The target molecule of the ADP-ribosylation reaction of pierisin-1 was DNA, which is completely different from other ADP-ribosylation toxins. If pierisin-1 can be endowed with selectivity for its potent cell-damaging activity, it may be possible to create powerful anticancer agents. Connecting cancer cell-specific receptor binding sites to the N-terminal side with cell-damaging activity or applying cutting-edge drug delivery system (DDS) technologies such as liposome formulation that select for cancer cells could make these possibilities a reality [86]. Moreover, pierisin-1 shows potent antifungal activity, especially against *Candida albicans*, and pierisin-1 could contribute to the creation of new antifungal drugs. Regarding antifungal drugs, no viruses infecting pathogenic fungi had been reported until recently. However, specific mycoviruses targeting pathogenic fungi have been discovered [87]. The utilization of such viruses may also contribute to the development of new drugs like phage therapy for bacteria [88,89].

The very potent cytotoxic and apoptosis-inducing activity of pierisin could also be applied to creating novel biological materials. In fact, it was reported that it was possible to establish transgenic silkworms with posterior silk glands that express the enzymatic domain of pierisin-1A. Pierisin-1A, a homolog of pierisin-1, had relatively lower DNA ADP-ribosylating activity than pierisin-1. Cocoons generated by the silkworms solely consisted of the glue-like glycoprotein sericin, from which soluble sericin could be prepared to form hydrogels [90].

Understanding of the biological nature of pierisin-1 with DNA ADP-ribosylating activity could provide informative data for the elucidation of the significance of pierisin-1 in cabbage butterfly, and this evidence could lead to the development of useful and novel biological materials. This may also be true in other pierisin-like proteins. Further research development is needed in the future. At such opportunity, Dr. Sugimura stated his impressions, “Nature is really and truly more complex and unknown than what is reported in papers and described in textbooks. We should treasure our deep interest in nature and keep a sharp eye for observation, which can lead us to interesting and novel discoveries”.

## Data Availability

The data presented in this study are available in this article and Supplementary Material here.

## Supplementary Materials

The following supporting information can be downloaded at: https://www.mdpi.com/article/10.3390/toxins16060270/s1. Figure S1: Effects of hormone agonists or analogs on pierisin-1 expression; Figure S2: The distribution of pierisin-1 proteins and their co-localization with apoptotic cells in the tissue of the white cabbage butterfly; Figure S3: Changes in pierisin-1 mRNA expression levels when bacteria were injected into first-day, third-instar larvae of the cabbage butterfly; Table S1: The effectiveness of pierisin-1 against microbes including bacteria and fungi.
