# Supplementary material for: Pierisin, Cytotoxic and Apoptosis-Inducing DNA ADP-Ribosylating Protein in Cabbage Butterfly
Source: Toxins (Basel). 2024 Jun 14;16(6):270. doi: 10.3390/toxins16060270 (PMC11209040; doi:10.3390/toxins16060270)
Supplement: Supplementary file 1 [file toxins-16-00270-s001.zip › toxins-2999815-supplementary.pdf]

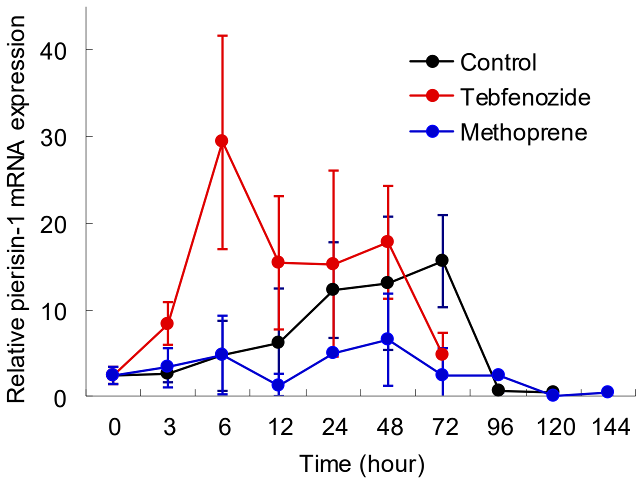

Figure S1. Effects of hormone agonists or analogs on pierisin-1 expression: Tebufenozide, an ecdysone receptor agonist (0.1 μg in 3 μl acetone), and Methoprene, a juvenile hormone agonist (3.75 μg in 3 μl acetone), were administered. A control group received 3 μl of acetone. Insect hormone agonists were injected into fifth instar larvae (1 day after molting), and mRNA expression levels were quantified using real-time RT-PCR.

Methods

Total RNA from the whole bodies of these host larvae was isolated using an RNeasy® Mini Kit (Qiagen). Synthesis of cDNA from total RNA was carried out with ReverTra Ace® and random primers (Toyobo, Japan) then real-time PCR was carried out in 96 -well plates with a 20 μl reaction volume containing 10 μl 2×SYBR® Green Realtime PCR Master Mix (Toyobo), 2 μM of each forward and reverse primer, and 1 μg of cDNA. The samples were subjected to denaturation at 95°C for 1 minute, followed by 40 cycles of amplification (95°C for 20 s, 60°C for 20 s and 72°C for 20 s) using DNA Engine Opticon2 (MJ Research, USA). Expression of pierisin-1 mRNA was normalized by 18S ribosomal RNA as an internal control. Values represent the average of 3-5 independent assays, and were statistically analyzed using the Student's t-test (Control vs hormone agonists). Results are presented as mean ± SD, with significance indicated by asterisks (*) with P<0.05. The primer combinations were as follows: 5′- CATAACGGACGCATTCAAAG -3′ (forward primer) and 5′- GGTTTTGGGTTATTAGGCTC -3′ (reverse primer) for pierisin-1 gene, 5′-ACAATTGGAGGGCAAGTCTG -3′ (forward primer) and 5′- CACCGCGATAGGATTTTGAT -3′ (reverse primer) for 18S ribosomal RNA gene.

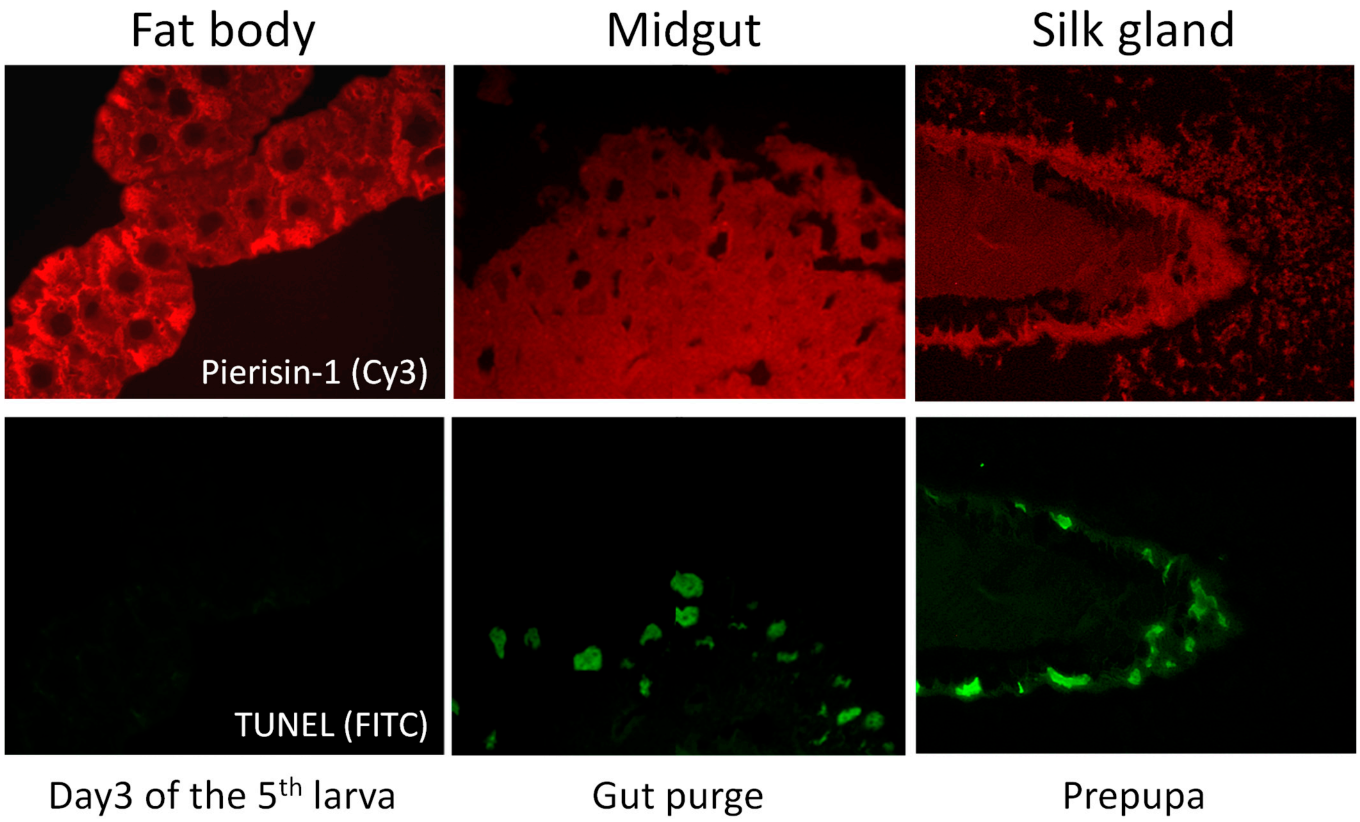

Figure S2. The distribution of pierisin-1 proteins and their co-localization with apoptotic cells in the tissue of the white cabbage butterfly. Pierisin-1 proteins are primarily synthesized in the fat body during the 5th instar larva stage. During the gut purge stage, which precedes the prepupal period, pierisin-1 proteins are internalized within cells undergoing apoptosis in the midgut. In the prepupal stage, co-localization of pierisin-1 proteins is observed in the silk gland, a tissue known to undergo apoptosis.

Methods

Formalin-fixed paraffin-embedded tissue sections of cabbage butterfly Pieris rapae were utilized. The sections were deparaffinized with xylene, and then hydrated with ethanol. Subsequently, the sections were rinsed with running water and subjected to microwave treatment at 500 W in 10 mM citrate buffer. Following this, the sections were incubated in 0.3% H2O2 methanol solution and washed with phosphate-buffered saline (PBS). Blocking was performed using Block Ace TM (Dainippon, Japan). Cy3-labeled anti-Pierisin-1 Rabbit IgG antibody [3] and 500 μl PBS were added, and the sections were left for 1.5 hours in a dark, moist tray. The TUNEL assay was conducted using the Apoptosis in situ detection kit (Wako) following the instruction manual, with the following modification. Afterward, they were washed and mounted with Vectashield mounting medium (Vector Laboratories, USA) for fluorescence. Subsequently, they were scanned using a Biozero standard fluorescence microscope (Keyence, Japan). In experiments involving antibody usage, we employed no primary antibody as the negative control. For the TUNEL assay, we utilized no TdT enzyme as the negative control. We conducted detection by fixing the exposure time during imaging when the negative controls were not visible.

Table S1 The effectiveness of pierisin-1 against microbes including bacteria and fungi.

			MIC (μg/ml)
Fungi	Yeast	*Candida albicans*	<0.063
		Cryptococcus neoformans	2
	Filamentous fungus	*Aspergillis niger*	8
		Trichophyton mentagrophytes	32<
Bacteria	Gram positive	*Micrococcus luteus*	50<
	Gram negative	*Escherichia coli*	50<

Methods

Fungal strains Candida albicans (SC5314), Cryptococcus neoformans (B4500), Aspergillus niger (CBS 554.65), and Trichophyton mentagrophytes (CBS 318.56) were stored at the Medical Mycology Research Center (MMRC), Chiba University. The bacteria Micrococcus luteus (NBRC13867) and Escherichia coli (JM109) were obtained from the NITE Biological Resource Center, Japan. Yeasts were cultured on YPD agar medium, filamentous fungi were cultured on PDA medium, and bacteria were cultured on LB medium. Yeasts, bacteria, and filter-extracted spores of filamentous fungi were suspended in RPMI 1640 medium (SIGMA)at a concentration of 10^3/ml, and the minimum inhibitory concentration (MIC) was measured against a half-dilution series of pierisin-1 for 48 hours.

Bacterial infection in P. rapae larvae increased the expression of pierisin-1 mRNA (Figure S3), but pierisin-1 itself had little effect on the bacteria (Table S1). However, it was highly effective against fungi, suggesting that pierisin-1 induces cell death by interfering with a cell entry pathway or an apoptosis pathway common to eukaryotic organisms.

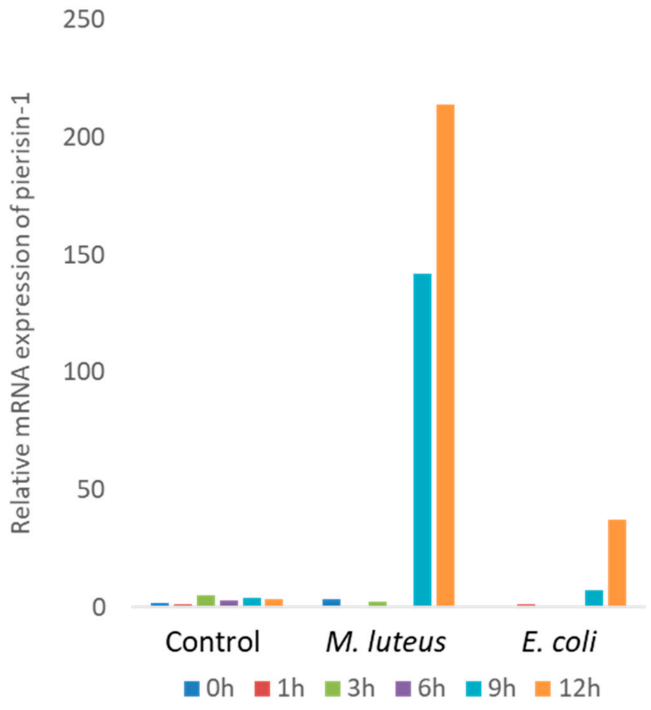

Figure S3. Changes in pierisin-1 mRNA expression levels when bacteria were injected into first-day, third-instar larvae of the cabbage butterfly.

Methods

Micrococcus luteus (NBRC13867) and Escherichia coli (JM109) were procured from NITE Biological Resource Center, Japan. Pieris rapae larvae weighing between 45 to 52 mg at the end of the 5th instar were used. Untreated individuals under the same conditions were designated as 0 hours post-treatment. Bacteria for injection experiments (M. luteus and E. coli) were cultured overnight in LB broth at 37°C. The cultures were then centrifuged at 13,000 rpm for 1 second to remove the supernatant, and the pellets were resuspended in PBS to achieve an optical density of 0.6 at 600 nm. Pieris rapae larvae at day 0 of the 5th instar were subjected to ice anesthesia for 10 minutes, and then 2 μl of various suspensions were injected into the hemocoel from the 6th to 7th abdominal segments using a Micro Dispenser (Drummond). PBS was used for the control group. After injection, the larvae were placed individually in Petri dishes lined with filter paper and incubated at 25±1°C in an incubator. Cabbage leaves were provided as food during the incubation period. After injection with bacteria and a specified time period, each larva was placed in a 1.5 ml tube and designated as a whole-body sample. The samples were stored at -80°C until RNA extraction. Total RNA of whole-body samples, the tissues were homogenized using a homogenizer pestle after adding Sepasol RNA I Super (Nacalai tesque, Japan). Realtime PCR methods were mentioned above (FigureS1).
